# Virological and Parasitological Characterization of Mini-LEWE Minipigs Using Improved Screening Methods and an Overview of Data on Various Minipig Breeds

**DOI:** 10.3390/microorganisms9122617

**Published:** 2021-12-18

**Authors:** Sabrina Halecker, Julia Metzger, Christina Strube, Ludwig Krabben, Benedikt Kaufer, Joachim Denner

**Affiliations:** 1 Institute of Virology, Freie Universität Berlin, 14163 Berlin, Germany; sabrina.halecker@fu-berlin.de (S.H.); Ludwig.Krabben@fu-berlin.de (L.K.); Benedikt.Kaufer@fu-berlin.de (B.K.); 2 Research Group Veterinary Functional Genomics, Max Planck Institute for Molecular Genetics, 14195 Berlin, Germany; jmetzger@molgen.mpg.de; 3 Institute of Animal Breeding and Genetics, University of Veterinary Medicine Hannover, 30559 Hannover, Germany; 4 Institute for Parasitology, Centre for Infection Medicine, University of Veterinary Medicine Hannover, 30559 Hannover, Germany; Christina.Strube@tiho-hannover.de

**Keywords:** minipigs, xenotransplantation, pig viruses, porcine endogenous retroviruses

## Abstract

Minipigs play an important role in biomedical research and have also been used as donor animals in xenotransplantation. To serve as a donor in xenotransplantation, the animals must be free of potential zoonotic viruses, bacteria and parasites. Porcine endogenous retroviruses (PERVs) are integrated in the genome of all pigs and cannot be eliminated as most of the other pig viruses can. PERV-A and PERV-B infect human cells in cell culture and are integrated in all pigs, whereas PERV-C infects only pig cells and it is found in many, but not all pigs. Minipigs are known for a high prevalence of recombinant PERV-A/C viruses able to infect human cells (Denner and Schuurman, *Viruses*, 2021;13:1869). Here, Mini-LEWE minipigs are screened for the first time for pig viruses including PERV. Peripheral blood mononuclear cells (PBMCs) from 10 animals were screened using PCR-based methods (PCR, RT-PCR, and real-time PCR). In comparison with our previous screening assays, numerous improvements were introduced, e.g., the usage of gene blocks as a PCR standard and foreign RNA to control reverse transcription in RT-PCR. Using these improved detection methods, Mini-LEWE pigs were found to be negative for porcine cytomegalovirus (PCMV), porcine lymphotropic herpesviruses (PLHV-1, -2 and -3), porcine circoviruses (PCV1, 2, 3 and 4), porcine parvovirus (PPV) and hepatitis E virus (HEV). All animals carried PERV-A, PERV-B and PERV-C in their genome. PERV-A/C was not found. In contrast to all other minipig breeds (Göttingen minipigs, Aachen minipigs, Yucatan micropig, Massachusetts General Hospital miniature pigs), Mini-LEWE minipigs have less viruses and no PERV-A/C. Parasitological screening showed that none of the Mini-LEWE minipigs harbored ecto- and gastrointestinal parasites, but at least one animal tested positive for anti-*Toxoplasma gondii* antibodies.

## 1. Introduction

Pigs (*Sus scrofa*) have great potential as biomedical models for studying developmental processes, congenital diseases and pathogen response mechanisms and can be used as xenotransplant organ donors and tools for vaccine and drug design [1,2]. Minipigs are also broadly used in several fields of biomedicine, e.g., experimental surgery, pharmacology, toxicology and basic research (for review see [3,4,5,6]). This particular group of miniature size pigs (called minipigs, micropigs or miniature swine) has moved into focus as a model animal with many well-documented similarities to humans. Various minipig breeds have been used for research purposes worldwide, including the Mexican Yucatan minipig or the Minnesota minipigs, two of the oldest breeds in laboratory use [3,7], or the Göttingen miniature pig (GöMP) developed in Europe [8]. An example of use of these breeds is the research work carried out for xenotransplantation, for which minipigs have been considered as potentially suitable donors of islet cells in order to alleviate the increasing shortage of human donors of pancreata [9]. Thus, a preclinical trial was performed using islet cells transplanted from Göttingen minipigs into non-human primates as a potential future treatment for diabetic patients [10]. These studies are part of the development of a new technology, xenotransplantation, using cells, tissues and organs from pigs in order to alleviate the increasing shortage of human donors [11]. Furthermore, preclinical trials have been performed to address possible complications that need to be considered for xenotransplantation and/or transplantation. In a study on lung transplantation, Mini-LEWE minipigs were used as a model for successful proof of concept that an ex vivo lung perfusion can act as a bioreactor for the rehabilitation of transplanted lungs [12].

This particular minipig population, named Mini-LEWE due to its original breeding locations Lehnitz and Wendefeld (LEWE) in the former German Democratic Republic (GDR), has been suggested as a genetic resource of versatile use as an animal model [13,14]. It was bred as a cross between Vietnamese Pot-bellied sows and a boar, which was derived from mating a German Saddleback pig with a Deutsches Veredeltes Landschwein [13,14]. Further backcrossing to Vietnamese Pot-bellied pigs and thorough selection finally resulted in a minipig of white skin, large ears with pronounced veins and the desired low body weight [15]. The Mini-LEWE pigs showed a similar diversity level when compared with the better known GöMP [14]. Mini-LEWE pigs represent a closed population, separated from historically related pig populations. A great genetic distance was found in Mini-LEWE to the GöMP as well as Leicoma and Large White [14]. Leicoma is a synthetic dam line breed also bred in the former GDR.

Using minipigs in different fields of biomedicine requires that the animals are healthy and do not suffer from infections, otherwise the results of the experiments will be falsified by the interaction of the microorganism with the animal. Transplantation and xenotransplantation studies using minipigs are especially vulnerable concerning microbiological infections, including virus infections. The reason is that the cells or organs will be transplanted into an immunosuppressed recipient. In the above-mentioned transplantation of islet cells from GöMP into non-human primates, no porcine viruses were transmitted [10]. The pigs used were derived from Ellegaard Göttingen Minipigs A/S, where they are bred at a very high hygienic standard. They tested negative for about 89 porcine microorganisms. However, some animals were positive for porcine cytomegalovirus (PCMV) [16], and hepatitis E virus (HEV) [17].

In other preclinical xenotransplantation trials using genetically modified donor pigs for orthotopic heart transplantation into baboons (*Papio anubis*), an infection of the donor pigs with PCMV resulted in a drastic reduction in the survival time of the xenotransplantat [18]. A similar reduction was observed when pig kidneys were transplanted into baboons or rhesus monkeys (*Macaca mulatta*) (for review, see [19]).

In addition to the GöMP, the Aachen minipigs were also screened for porcine viruses [20,21]. HEV RNA was found by real-time reverse transcriptase PCR (real-time RT-PCR) in most, and DNA of PCMV, PLHV-2, and PLHV-3 was found by PCR in some animals. The animals were free of eight other microorganisms tested, but some were seropositive for porcine circovirus 2 (PCV2), porcine reproductive and respiratory syndrome virus (PRRSV), and porcine epidemic diarrhea virus (PEDV).

Porcine endogenous retroviruses pose a special risk for xenotransplantation, because they are integrated into the genome of all pigs and cannot be eliminated as all other viruses can. PERV-A and PERV-B were found in all pigs, they are able to infect human cells in cell culture [22]. PERV-C is present in most, but not all pigs, and it infects only pig cells [23]. Recombinations between PERV-A and PERV-C were observed in vivo, in the living pigs, and these PERV-A/C viruses are able to infect human cells and they are characterized by high replication rates (for review see [24]). In GöMP from Ellegaard Göttingen Minipigs A/S, Denmark [16] and from the University of Göttingen [25] all three PERVs were found integrated in the genome. Whereas in a small number of Ellegaard GöMPs no PERV-A/C were found, in three out of 11 animals from the University of Göttingen PERV-A/C was found. In one case an infectious PERV-A/C isolate able to infect human 293 cells was released [25]. Interestingly, the prevalence of PERV-A/C is generally high in minipigs (for review see [26]). There is consensus that only PERV-C-free pigs should be used for xenotransplantation in order to avoid PERV-A/C recombination [27]. Whether it will be necessary and possible to use pigs treated with CRISPR/Cas9 to inactivate all PERV copies in the genome [28,29] is still unclear [30].

Whereas pigs are well characterized concerning their viruses, the analysis of bacteria and parasites has been less comprehensive. In most cases only viruses [20,25,31], in some cases also bacteria [16,32], and only in rare cases also parasites were studied [33]. Here, we perform for the first time a broad virological and parasitological analysis of the Mini-LEWE pigs.

## 2. Materials and Methods

### 2.1. Mini-LEWE: Genetics and Breeding Conditions

All today’s breeding animals are derived from 5 boars and 10 sows born in 2003/2004, which were the only ones left with the demise of former GDR [14]. They were the last remnants from a breeding program started in 1965 at Humboldt University in Berlin [34], in which Vietnamese Pot-bellied sows were initially mated with a white boar (German Saddleback X Deutsches Veredeltes Landschwein) and their progeny subsequently backcrossed using Vietnamese Pot-bellied pigs [13]. This population kept a surprisingly nearly constant level of genetic diversity in a similar fashion as the more frequently used population of GöMP [14]. Nevertheless, the Mini-LEWE are genetically distinct to historically related breeds including GöMP, Leicoma and Large White [14].

The Mini-LEWE population is kept now under conventional farm-like (not specified pathogen free) conditions at the Farm for Education and Research Ruthe at a remote location of the University of Veterinary Medicine Hannover. In total, 14 breeding animals and 47 offspring represent the current Mini-LEWE population. A breeding pro-gram was performed within this closed population aiming at long generation intervals with low relationship coefficients to keep the increase in inbreeding as low as possible [35]. The average inbreeding coefficient was estimated to be 11.7% [35]. All individuals were kept on untreated (not sterilized) straw and fed with grain mixture (wheat meal, wheat bran) supplemented with mineral feed. They underwent a standard health monitoring, but despite current investigations, were not investigated by special screenings for viruses, bacteria, fungi, or parasites.

### 2.2. Samples and Mitogen Stimulation of Peripheral Blood Mononuclear Cells

To determine the virological status of the Mini-LEWE minipigs, heparin blood samples taken from ten minipigs for diagnostic purposes (Table 1) were investigated. For the isolation of peripheral blood mononuclear cells (PBMCs), the heparin blood was dispensed on Pancoll human (PAN-Biotech GmbH, Aidenbach, Germany), centrifugated at 900 g for 21 min and the isolated PBMCs were washed twice with phosphate-buffered saline. One million cells per sample were diluted in 2 mL Roswell Park Memorial Institute medium (RPMI-1640, PAN-Biotech GmbH, Aidenbach, Germany), seeded in a 12-well plate (Sarstedt AG & Co. KG, Nümbrecht, Germany) and stimulated with 10 µg/mL phytohemagglutinin-L (PHA-L) Solution (500 ×) (Invitrogen, Waltham, MA, USA). After five days, the activated PBMCs were pelleted and stored at −20 °C until further processing.

### 2.3. DNA and RNA Isolation

DNA nucleic acid extraction from activated PBMCs was carried out using the DNeasy Blood and Tissue kit (Qiagen, Hilden, Germany) for the detection of PCMV, PLHV-1, PLHV-2, PLHV-3, PCV-1, PCV-2, PCV-3, PCV-4, PPV-1 and to determine the proviral status of PERV A, B and C. For the detection of HEV, total RNA was extracted from activated PBMCs using the RNeasy Mini kit (Qiagen, Hilden, Germany) according to the manufacturer’s instructions in an elution volume of 30 µL. For the quantification of DNA and RNA in all samples, a NanoDrop Spectrophotometer ND-1000 (peqlab Biotechnologie GmbH, Erlangen, Germany) was used and the samples were stored at −20 °C until further processing.

### 2.4. PCR and Real-Time RT-PCR for the Detection of Porcine Endogenous Retroviruses

For the detection of all three subtypes, PERV-A, PERV-B and PERV-C, a real-time PCR targeting the PERV polymerase (PERV pol) was carried out using a TaqMan-based PCR (Table 2) [36] and using the SensiFAST Probe No-ROX kit (Meridian Bioscience, Cincinnati, OH, USA) in a reaction volume of 20 µL. Real-time PCR was performed on the qPCR cycler qTOWER^3^ G (Analytik Jena, Jena, Germany) using a temperature–time profile that comprises an activation step of 5 min at 95 °C, followed by 45 cycles of 15 s at 95 °C for denaturation, 30 s at 58 °C for annealing and 30 s at 72 °C for elongation.

To detect PERV-C, a conventional PCR was carried out, using specific primers for the envelope gene of PERV C [37]. This real-time PCR was carried out in a reaction volume of 25 µL comprising 1.2 µL of 10 µM env.2-forward primer, 1.2 µL of 10 µM env.2-reverse primer, 0.125 µL AmpliTaq DNA Polymerase (Applied Biosystems, Waltham, MA, USA), 2.5 µL of 10× PCR Buffer I (Applied Biosystems, Waltham, MA, USA) and 0.5 µL of 10 mM dNTP Mix (Thermo Fisher Scientific, Waltham, MA, USA) and applied on the Biometra TRIO cycler (Analytik Jena, Jena, Germany).

To detect PERV-A/C [38], a PCR was performed in a reaction volume of 25 µL containing 1 µL of 10 µM PERV-A env VRBF, 1 µL of 10 µM PERV-C env TMR, 0.125 µL AmpliTaq DNA Polymerase (Applied Biosystems, Waltham, MA, USA), 2.5 µL of 10x PCR Buffer I (Applied Biosystems, Waltham, MA, USA), 0.5 µL of 10 mM dNTP Mix (Thermo Fisher Scientific, Waltham, MA, USA) and filled to a final volume of 25 µL with nuclease-free water. The PCR was run on the Biometra TRIO cycler (Analytik Jena, Jena, Germany).

### 2.5. Real-Time RT-PCR for the Detection of Hepatitis E Virus

For the detection of HEV, a TaqMan based real-time RT-PCR described by Jothikumar et al. was performed [39]. The oligosequences of the primers and probes are listed in Table 2. All real-time RT-PCR reactions were prepared using the SenisFAST™ Probe No-ROX One-Step Kit (Meridian Bioscience, Cincinnati, OH, USA) in a volume of 16 µL and the real-time RT-PCR was performed at the qPCR cycler qTOWER^3^ G (Analytik Jena, Jena, Germany). The temperature time profile applied consisted of a reverse transcriptase step of 30 min at 50 °C and an activation step of 15 min at 95 °C, followed by 45 cycles of 10 s at 95 °C, 20 s at 55 °C and 15 s at 72 °C. To further enhance the reliability of the assay and to check the presence of inhibitory substances, an internal control (IC) system was integrated and validated. Two microliters of Influenza A-RNA derived from a mouse-adapted Influenza A strain A/WSN/1933 were spiked to each sample during the lysis of the nucleic acid extraction. The IC was detected by using a Flu-A (NA) real-time RT-PCR assay [40] that was applied as a duplex real-time RT-PCR assay along with the detection of the gene of interest.

### 2.6. PCR and Real-Time PCR for the Detection of DNA Viruses

The detection of PCMV, PLHV-1, PLHV-2, PLHV-3 and PCV1, PCV2, PCV3, PCV4 and PPV-1 was performed by species-specific real-time PCR assays (Table 2) based on TaqMan technology as described previously [37,41,42,43,44,45]. All experiments were performed with the SensiFAST Probe No-ROX kit (Meridian Bioscience, Cincinnati, OH, USA) at the qPCR cycler qTOWER^3^ G (Analytik Jena, Jena, Germany). The fluorescence signal was measured during the annealing step. All assays were performed as duplex real-time PCR using the reference gene porcine glycerinaldehyd-3-phosphat-dehydrogenase (pGAPDH), which is also an excellent reference gene in expression studies [46,47].

For the PCMV real-time PCR [41], a reaction volume of 20 µL was prepared containing 1.8 µL of PCMV-FAM mix and 1.8 µL of pGAPDH-HEX mix as IC and 4.0 µL of extracted DNA. The reaction was carried out for 2 min at 50 °C for activation 10 min at 95°C followed by 45 cycles comprising 15 s at 95 °C for denaturation and 60 s at 60 °C for annealing and elongation.

PLHV-1, -2 and 3 were detected as single real-time PCR, each performed in a 20 µL reaction volume as described by Chmielewicz et al. [42]. The temperature–time profile applied consisted of an activation step of 5 min at 95 °C, followed by 45 cycles containing 15 s at 95 °C for denaturation, 60 s at 56 °C for annealing and 30 s at 72 °C for extension.

For the detection of four different PCVs, independent real-time PCR with a reaction volume of 20 µL was performed. The real-time PCR for PCV1, PCV2 and PCV4 was carried out as described by Chen et al. [43], while the real-time PCR assay for PCV3 was performed according to the recommendations of Palinski et al. [44]. Each probe was labeled with a 6-carboxyfluorescein (6-FAM) fluorophore for PCV-1, PCV-2, PCV-3 and PCV-4, whereas the probe for pGAPDH was labeled with a HEX fluorophore [43].

For the PPV-1 real-time PCR [45], a reaction volume of 20 µL was prepared including 1.8 µL of PPV1-FAM mix and 1.8 µL of pGAPDH-HEX mix and 4.0 µL of extracted DNA. The PCR run was performed for 5 min at 95 °C, followed by 40 cycles comprising 15 s at 95 °C, 60 s at 56 °C and 30 s at 72 °C.

### 2.7. Generation of Viral Standards

**Figure 1:**
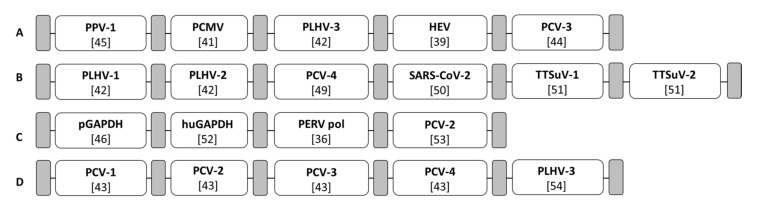
Schematic representation of four gBlocks: gBlock I (**A**), gBlock II (**B**), gBlock III (**C**), gBlock IV (**D**). Sequences of the investigated viruses were taken from [36,39,41,42,43,44,46,49,50,51,52,53,54]. Grey boxes indicate spacers; white boxes are virus-specific oligosequences corresponding to primer and probe. References are given in brackets; detailed information on the oligosequences are listed in Table S1.

For validation purpose and optimization of the real-time PCR used, four different gBlock Gene Fragments (hereinafter referred as gBlock; Integrated DNA Technologies, IDT, Coralville, IA, USA) were designed [48]. gBlocks are synthetic double-stranded DNA fragments, which can be used as a standard for a various number of applications in molecular biology. In this study, the gBlocks comprise the nucleotide sequence of the genes of interest, which are separated by spacers (Figure 1). The sequences of the investigated viruses were taken from [36,39,41,42,43,44,46,49,50,51,52,53,54]. The spacers are non-functional oligosequences of eight to fifteen base pairs that are used as placeholders to separate the oligosequences of interest. The nucleotide sequence of the gBlocks were submitted to IDT for production and access control. The lyophilized gBlocks were dissolved according to the manufacturer’s instructions and the DNA content was measured using a Qubit 4 Fluorometer (Thermo Fisher Scientific, Waltham, MA, USA). The copy number/µL can be calculated according to the formula [48]:

$$copy\;number\;per\;µL=c\times M\times 1\times 10^{-15}\;\frac{\mathrm{mol}}{f\mathrm{mol}}\times \;\mathrm{Avogadro}\prime s\;\mathrm{number}\;$$

where *c* is the current concentration of the gBlock in ng/µL, and *M* is the molecular weight in fmol/ng. Afterwards, a log_10_ dilutional series was prepared in nuclease-free water. In the case of positive samples, a standardization was performed by application of the log_10_ dilutional series of the corresponding gBlocks containing the gene of interest and pGAPDH, respectively (Figure S1).

### 2.8. Minimum Information for Publication of Quantitative Real-Time PCR Experiments (MIQE)

For an improved reporting of technical information concerning the qPCR approaches used in this study, experimental details were provided in accordance with the MIQE guidelines [55,56]. In this study, the qPCR assays are well-established diagnostic methods (see Table 2) that were adapted to the requirements in xenotransplantation. Quality control tools and measurements were conducted to ensure reliable and reproducible test results in diagnostic applications (Tables S1 and S2).

### 2.9. Parasitological Screening

All animals were carefully checked for macroscopically visible ectoparasites (lice, ticks). To test for sarcoptic mange infestations, sera of the individual pigs were checked for antibodies using the Sarcoptes-ELISA 2001 Pig (Afosa, Blankenfelde-Mahlow, Germany) according to the manufacturer’s instructions. Individual serum samples were also used to screen for Toxoplasma gondii infection with the ID Screen Toxoplasmosis Indirect Elisa (IDvet, Grabels, France), as recommended by the manufacturer. Accordingly, samples with a sample/positive (S/P) ratio ≤ 40% were classified as negative, with 40% < S/P < 50% as questionable and with S/P ≥ 50% as positive.

Infections with gastrointestinal parasites were tested by fecal examination. To consider potential intermittent egg, oocyst or cyst shedding, all pigs were sampled on three consecutive days. All fecal samples (*n* = 30) were processed individually using the combined sedimentation–flotation technique as previously described [57].

## 3. Results

### 3.1. Prevalence of Porcine Endogenous Retroviruses

To determine the presence and proviral status of PERV, a set of PCRs, and real-time PCRs were carried out analyzing nucleic acids extracted from mitogen-stimulated and unstimulated PBMCs. The ten Mini-LEWE minipigs were positive for the polymerase gene (pol) of PERV, indicating that PERV is present in the genome of the Mini-LEWE minipigs as expected. PERV-A and PERV-B are present in all pig breeds. A conventional PCR, which uses a primer pair targeting the envelope gene (env) of PERV-C, revealed that in addition all animals were PERV-C positive (Table 3). Since the presence of PERV-C is a prerequisite for a recombination event to generate PERV A/C recombinants, all animals were tested for PERV A/C. All Mini-LEWE minipigs were negative for PERV A/C.

### 3.2. Screening for Hepatitis E Virus

Screening for hepatitis E virus (HEV), a porcine RNA virus with known zoonotic potential, was performed with a real-time RT-PCR and all samples were found to be negative (Table 4).

To improve the HEV real-time RT-PCR, an internal control (IC) system was established to check the RNA isolation and the reverse transcription reaction. As IC, a real-time PCR detecting the neuraminidase (NA) gene of the influenza virus-A strain A/WSN/1933 (abbreviated Flu-A (NA)) was used. With the help of a log_10_ dilutional series of the Flu-A virus-containing cell culture supernatant diluted in nuclease-free water, an optimal concentration was found, which was added to each minipig sample before the isolation of the RNA.

### 3.3. Screening for Porcine Herpes Viruses, Circoviruses and Porcine Parvovirus-1

The Mini-LEWE pigs were screened for four different herpes viruses (PCMV, PLHV-1, PLHV-2, PLHV-3), four circoviruses (PCV1, PCV2, PCV3, PCV4) and porcine parvovirus-1 (PPV-1); all animals were negative.

### 3.4. Establishment of gBlocks for the Standardization

For an easy-to-handle and less time-consuming preparation of a real-time PCR-standard, four gBlocks were designed (Figure 1). Each gBlock contains specific oligosequences corresponding to the PCR amplicon of the gene of interest of different virus species (Table S3), which are divided by spacers. After dissolution of the lyophilized gBlocks, the DNA content was determined by using the Qubit 4 Fluorometer. The DNA content measured varied between 1.13 and 4.72 ng/µL (Table S1) and was a prerequisite for the calculation of the copy numbers of the gBlock stock solutions. The gBlock stock solutions comprised 1.6 to 7.4 × 10^9^ copies/µL (Table S1). Afterwards, the gBlock stock solution was diluted in a log_10_ dilutional series (10^8^ to 10^−1^) per gBlock and the dilutional steps (starting with 10^6^) were applied in a corresponding real-time PCR run. A standard curve was calculated by the qPCRsoft software package of Analytik Jena (Jena, Germany) by which the cycle threshold (Ct) values are plotted against the *copy numbers*/µL and the PCR efficiencies were calculated. The PCR efficiencies ranged from 0.85 to 1.07 (Table S2). The log_10_ dilutional series of all gBlocks showed a similar Ct value at the same dilution step, indicating that the run conditions for each real-time PCR assay are optimized. Since all animals were negative for all tested viruses, there was no need to estimate the copy number.

### 3.5. Screening for Pig Parasites

None of the ten tested Mini-LEWE pigs were infested with large ectoparasites such as lice or ticks and all individuals tested serologically negative for sarcoptic mange mites. Furthermore, all animals tested coproscopically negative for eggs, oocysts or cysts of gastrointestinal parasites. Screening for anti-Toxoplasma gondii antibodies revealed the animal with the sample ID LEWE-3 as seropositive (S/P = 75%), while LEWE-2 was questionable (S/P = 47%).

## 4. Discussion

This is the first virological examination of the Mini-LEWE minipigs at the molecular level using a set of PCR-based methods. When the animals were first characterized at the end of the 1970s, the clinical absence of virus infection was mainly diagnosed, and no molecular and only one immunological assay had been performed [58]. At that time Gregor et al. [58] reported that the animals kept under specified pathogen-free conditions were serologically free of brucellosis, caused by Brucella bacteria; transmissible gastro-enteritis (TGE), caused by the coronavirus TGEV; leptospirosis, caused by *Leptospira* bacteria; Aujeszky’s disease, caused by the Suid alphaherpesvirus 1 (SuHV-1), also called pseudorabies virus; and that the animals were clinically free of dysentery; salmonellosis, caused by *Salmonella* bacteria; Teschen disease and Talfan disease, both caused by teschoviruses; swine vesicular disease, caused by the swine vesicular disease virus, an enterovirus; foot-and-mouth disease, caused by the foot-and-mouth disease virus, an aphthovirus; classical swine fever caused by pestivirus C; porcine enzootic pneumonia, caused by *Mycoplasma hyopneumoniae*; pleuropneumonia due to *Haemophilus pleuropneumoniae*; and rhinitis atrophicans, caused by *Pasteurella multocida*. Serological tests showed the absence of pig-pathogenic mycoplasms, *Chlamydia*, *Bordetella bronchiseptica* and *Pasteurella multocida*. Furthermore, the animals were free of lice, mange mites and endoparasites.

In this study, nine DNA viruses (PCMV, PLHV-1, PLHV-2, PLHV-3 and PCV1, PCV2, PCV3, PCV4 and PPV-1), one RNA virus (HEV) and the integrated PERV proviruses (PERV-A, PERV-B, PERV-C and PERV-A/C) were analyzed because these viruses were declared as xenotransplantation relevant [25,59]. Although it still remains unclear, which porcine virus may cause a zoonosis when transmitted to human xenotransplant recipients, HEV is a well-known zoonotic virus which can be transmitted from pigs by undercooked pig meat and direct contact with infected animals (for review see [60]). Transmission of HEV from infected humans to other humans by blood transfusion and organ transplantation has also been observed (for review see [61]). HEV causes self-limiting acute hepatitis, acute liver failure, and neurological illness. Immunosuppressed individuals are at risk of developing chronic infections which may lead to liver fibrosis and cirrhosis.

The second virus with known zoonotic potential, at least for non-human primates, is PCMV, which is actually a porcine roseolovirus (PRV), closely related to the human herpes viruses 6A, 6B and 7. Transmission of PCMV/PRV in preclinical trials resulted in a significant reduction in the survival time of pig kidneys and hearts in rhesus monkeys and baboons (for review see [19]). In a recent study, orthotopic pig heart transplants survived up to 195 days in baboons, whereas the transplants from PCMV/PRV-positive animals survived less than 30 days [18]. PCMV/PRV was shown to increase the levels of interleukin-6 (IL-6) and tumor necrosis factor α (TNFα) in the transplanted baboons. In addition, increased levels of tissue plasminogen activator (tPA) and plasminogen activator inhibitor 1 complexes (tPA-PAI-1) were found, as measured in plasma samples by an ELISA. This indicates a complete loss of the pro-fibrinolytic properties of the endothelial cells. Since a reduction in the transplant survival time was observed in two different non-human primates (baboons, rhesus monkeys), it is very likely that the same may happen in human recipients.

Although transmission of PCV3 in a preclinical trial transplanting pig hearts into baboons was demonstrated, no pathological effects in the recipients were observed [62]. There are no reports showing transmission of any of the other viruses to non-human primate recipients. This is also true for PERVs, which are integrated into the genome of all pigs and therefore cannot be eliminated easily. PERV was also not transmitted in the first clinical trials transplanting pig islet cells in diabetic patients in New Zealand [63] and Argentina [64], as well as in more than 200 other human xenotransplant recipients (for review see [65,66]).

It is important to note that in this study only PBMCs isolated from fresh blood of the Mini-LEWE pigs were analyzed. It is possible that some viruses, especially latent viruses, may hide in certain organs and were not detected in the blood. For example, in one preclinical trial transplanting a pig heart orthotopically into baboons, PCMV was not detected in the blood of the donor pig, but transmitted to the recipient baboon [67]. Furthermore, microorganisms may also interact and one microorganism may enhance the effect of other microorganisms and worsen the clinical score associated with the disease [68].

Nevertheless, among all minipigs analyzed in our laboratory, the Mini-LEWE pigs have less exogenous viruses and surprisingly for minipigs have no PERV-A/C, which is common in minipigs [26] (Table 5). Minipigs that we did not analyze, such as the Massachusetts General Hospital SLA-defined miniature pigs, are also characterized by a high prevalence of PERV-A/C [38,69] and the presence of PCMV [70,71].

To test the viruses by PCR or real-time PCR, three new improvements were introduced. First, the usage of a Qubit instead of the less sensitive Nanodrop, second, the use of a control RNA virus, here the use of influenza virus RNA, to demonstrate that the transcription of the RNA by the reverse transcriptase took place in all tubes identical, and third, the use of gBlocks. The gBlocks guarantee a reproducible positive control on one hand, and on the other hand they will allow an exact determination of the copy number in the case virus is found. These improvements will guarantee a more correct testing in future.

Whereas in most cases the donor pigs were tested only for viruses [20,25,31], or viruses and bacteria [16,32], one study also screened for parasites [33]. The authors screened for pathogenic protozoa including *Cryptosporidium parvum*, *Giardia* sp. and *Toxoplasma gondii*, helminths, *Trichinella spiralis*, blood parasites and pathogenic arthropods such as lice and mites. In the present study, we examined the Mini-LEWE minipigs for common pathogenic parasites. No ectoparasites such as lice, ticks or zoonotic sarcoptic mites were detected. Furthermore, fecal examination to detect eggs, oocysts or cysts of gastrointestinal parasites, e.g., of (zoonotic) helminths such as *Ascaris suum*, *Strongyloides ransomi* or *Trichuris suis*, or (zoonotic) protozoans such as *Giardia* spp., *Cystoisospora* (former: *Isospora*) *suis* or *Eimeria* spp., was negative in all individuals. However, when testing for *Toxoplasma gondii* infections, one animal tested seropositive, and another one questionable. *T. gondii* is an important opportunistic pathogen in immunocompromised patients and can be transmitted from animals to humans with, e.g., blood products or tissue transplants [74]. In the present study, Mini-LEWE minipigs were housed under conventional conditions and kept on straw, providing various possibilities for *T. gondii* exposure. Conventional housing involves the risk of (infective) parasite stages being introduced by staff or equipment. Straw bedding in pig farming and laboratory husbandry provides employment and nest-building material, thus promoting animal welfare. If properly dried, straw is not a particular microbial hygiene risk; however, *T. gondii* infections can occur if animals have access to the storage rooms and the straw is contaminated with cat feces or dead rodents [75]. Although toxoplasmosis can be treated with antiparasitic drugs such as pyrimethamine, spiramycine and sulfadiazine, plus folinic acid, cysts are not eliminated completely. Therefore, animals generated for xenotransplantation should be tested regularly by serological methods and negative animals should be kept under designated pathogen-free conditions.

## 5. Conclusions

Mini-LEWE minipigs generated originally in East Germany were screened for the first time for several xenotransplantation-relevant pig viruses, including PERV, using PCR-based methods. All animals were found to be negative for PCMV, PLHV-1, -2 and -3, PCV1, 2, 3 and 4, PPV-1 and HEV. All animals carried PERV-A, PERV-B and PERV-C in their genome, but recombinant PERV-A/C was not found. In contrast to all other minipig breeds tested in the past, such as Göttingen minipig, Aachen minipig, Yucatan minipig or Minnesota minipigs, Mini-LEWE minipigs have less viruses and no PERV-A/C. *Toxoplasma gondii*, which may pose a high risk in immunosuppressed humans, was found in at least one animal, suggesting that a screening for parasites should also be performed in animals which may be used in xenotransplantation.

## Figures and Tables

**Table 1:** Characterization of the ten Mini-LEWE minipigs that were tested in this study.

Sample ID	Gender	Animal Status	Age at the Time of Sampling
LEWE-1	female	breeding animal	10 years
LEWE-2	female	breeding animal	6 years
LEWE-3	female	breeding animal	5 years
LEWE-4	male	breeding animal	6 months
LEWE-5	male	offspring	13 months
LEWE-6	male	offspring	13 months
LEWE-7	male	offspring	6 months
LEWE-8	male	offspring	6 months
LEWE-9	male	offspring	4 months
LEWE-10	male	offspring	4 months

**Table 2:** Primers and probes used in this study.

PCR Assay	Primer/Probe	Sequence 5′-3′	Amplicon (Base Pair)	Reference
HEV	JVHEV-Fwd	GGT GGT TTC TGG GGT GAC	70	[39]
	JVHEV-Rev	AGG GGT TGG TTG GAT GAA		
	JVHEV-Probe	6FAM-TGA TTC TCA GCC CTT CGC-BHQ		
PCMV	PCMV-Fwd	ACT TCG TCG CAG CTC ATC TGA	63	[41] modified
	PCMV-Rev	GTT CTG GGA TTC CGA GGT TG		
	PCMV-Probe	6FAM-CAG GGC GGC GGT CGA GCT C-BHQ		
PLHV-1	PLHV-1 (1125)-Fwd	CTC ACC TCC AAA TAC AGC GA	73	[42]
	PLHV-1 (1125)-Rev	GCT TGA ATC GTG TGT TCC ATA G		
	PLHV-1 (1125)-Probe	6FAM-CTG GTC TAC TGA ATC GCC GCT AAC AG-TAMRA		
PLHV-2	PLHV-2 (1155)-Fwd	GTC ACC TGC AAA TAC ACA GG	74	[42]
	PLHV-2 (1155)-Rev	GGC TTG AAT CGT ATG TTC CAT AT		
	PLHV-2 (1155)-Probe	6FAM-CTG GTC TAC TGA AGC GCT GCC AAT AG-TAMRA		
PLVH-3	PLHV-3 (1156)-Fwd	CTC ACC TCC AAA TAC AGC GA	73	[42]
	PLHV-3 (1156)-Rev	GCT TGA ATC GTG TGT TCC ATA G		
	PLHV-3 (1156)-Probe	6FAM-CTGGTCTACTGAATCGCCGCTAACAG-TAMRA		
PCV1	PCV-1 (F2020)-Fwd	AAC CCC ATA AGA GGT GGG TGT T	129	[43] modified
	PCV-1 (F2020)-Rev	TTC TAC CCT CTT CCA AAC CTT CCT		
	PCV-1 (F2020)-Probe	6FAM-TCC GAG GAG GAG AAA AAC AAA ATA CGGGA-BHQ1		
PCV2	PCV-2 (F2020)-Fwd	CTG AGT CTT TTT TAT CAC TTC GTA ATG GT	146	[43] modified
	PCV-2 (F2020)-Rev	ACT GCG TTC GAA AAC AGT ATA TAC GA		
	PCV-2 (F2020)-Probe	6FAM-TTA AGT GGG GGG TCT TTA AGA TTA AAT TCT CTG AAT TGT-TAMRA		
PCV3	PCV-3-Fwd	AGT GCT CCC CAT TGA ACG	112	[44]
	PCV-3-Rev	ACA CAG CCG TTA CTT CAC		
	PCV-3-Probe	6FAM-ACC CCA TGG CTC AAC ACA TAT GAC C-BHQ1		
PCV4	PCV-4 (F2020)-Fwd	ATT ATT AAA CAG ACT TTA TTT GTG TCA TCA CTT	103	[43]
	PCV-4 (F2020)-Rev	ACA GGG ATA ATG CGT AGT GAT CAC T		
	PCV-4 (F2020)-Probe	6FAM-ATA CTA CAC TTG ATC TTA GCC AAA AGG CTC GTT GA-BHQ1		
PPV-1	PPV-1-Fwd	CAG AAT CAG CAA CCT CAC CA	106	[45] modified
	PPV-1-Rev	GCT GCT GGT GTG TAT GGA AG		
	PPV-1-Probe	6FAM-TGC AAG CTT/ZEN/AAT GGT CGC ACT AGA CA-BHQ1		
PERVpol	PERVpol-Fwd	CGA CTG CCC CAA GGG TTC AA	236	[36]
	PERVpol-Rev	TCT CTC CTG CAA ATC TGG GCC		
	PERVpol-Probe	6FAM-CAC GTA CTG GAG GAG GGT CAC CTG-BHQ1		
PERV-C	envC.2-Fwd	GAT TAG AAC TGG AAG CCC CAA GTG CTC T	288	[37]
	envC.2-Rev	TCT GAT CCA GAA GTT ATG TTA GAG GAT GGT		
PERV-A/C	PERV-A env VRBF-Fwd	CCT ACC AGT TAT AAT CAA TTT AAT TAT GGC	1266	[38]
	PERV-C env TMR-Rev	CTC AAA CCA CCC TTG AGT AGT TTC C		
pGAPDH	pGAPDH-Fwd	ACA TGG CCT CCA AGG AGT AAG A	106	[46]
	pGAPDH-Rev	GAT CGA GTT GGG GCT GTG ACT		
	pGAPDH-Probe	HEX-CCA CCA ACC CCA GCA AGA GCA CGC-BHQ1		
Flu-A (NA)	NG05 NA-Fwd	CTG GAC TAG TGG GAG CAT CA	93	[40] modified
	NG06 NA-Rev	ATG GTG AAC GGC AAC TCA G		
	NG07 NA-Probe	HEX-CAC CGT CTG GCC AAG ACC AAT C-BHQ1		

Fwd = forward primer, Rev = reverse primer.

**Table 3:** Prevalence of PERVs in Mini-LEWE minipigs ^1^.

Sample ID	PERVpol	PERV-C	PERV-A/C
LEWE-1	15.12	positive	negative
LEWE-2	14.47	positive	negative
LEWE-3	15.37	positive	negative
LEWE-4	16.14	positive	negative
LEWE-5	14.01	positive	negative
LEWE-6	15.31	positive	negative
LEWE-7	15.43	positive	negative
LEWE-8	17.49	positive	negative
LEWE-9	16.01	positive	negative
LEWE-10	16.70	positive	negative

^1^ PERV pol were detected using a real-time PCR, and the numbers indicate the Ct value; PERV-C and PERV-A/C were detected using a conventional PCR.

**Table 4:** Absence of HEV in ten Mini-LEWE minipigs.

Sample ID	HEV	Flu-A (NA)
LEWE-1	no Ct	27.20 *
LEWE-2	no Ct	26.59
LEWE-3	no Ct	26.83
LEWE-4	no Ct	27.23
LEWE-5	no Ct	32.91
LEWE-6	no Ct	31.06
LEWE-7	no Ct	31.83
LEWE-8	no Ct	27.60
LEWE-9	no Ct	32.17
LEWE-10	no Ct	26.92

* The numbers indicate the Ct values.

**Table 5:** Virus prevalence in six minipig breeds analyzed in our laboratory.

Minipig Breed ^1^	Institution/ Company	PERV-C	PERV-A/C	PCMV	PCV1	PCV2	PCV3	HEV	PLHV-1	PLHV-2	PLHV-3	References
Mini LEWE ^2^	University of Veterinary Medicine Hannover, Germany	10/10 PBMCs	0/10 PBMCs	0/10 PBMCs	0/10 PBMCs	0/10 PBMCs	0/10 PBMCs	0/10 PBMCs	0/10 PBMC	0/10 PBMCs	0/10 PBMCs	this manuscript
Göttingen minipigs	Ellegaard Göttingen Minipigs A/S, Denmark	28/28	None PBMCs	10/22 PBMC liver spleen	n.t. ^3^	3/21 PBMC liver spleen	0/10 PBMC liver spleen	9/40 serum	1/10 anti-bodies	0/5 PBMC liver spleen	0/5 PBMC liver spleen	[10,16,17]
Göttingen minipigs	University Göttingen, Göttingen, Germany	11/11	3/13 PBMCs	PBMCs 0/10	n.t.	3/10 PBMCs	0/10 PBMCs	0/10 PBMCs	2/11 PBMCs	2/11 PBMCs	2/11 PBMCs	[25]
Aachen minipigs ^4^	Aachen Minipig, Heinsberg, Germany	18/18	3/18 liver spleen	5/18 PBMCs	n.t.	6/18 antibodies	n.t.	12/18 serum	0/18 PBMCs	5/18 PBMCs	2/18 PBMCs	[20]
Yucatan micropig	Charles River, Saint-Aubin-Les-Elbeuf, France	1/1 ^5^	1/1 PBMCs	n.t.	n.t.	n.t.	n.t.	n.t.	n.t.	n.t.	n.t.	[72]
Munich miniature swine (MMS) ^6^	Institute of Veterinary Pathology, University of Munich	Yes	5/5 PBMCs	n.t.	n.t.	n.t.	n.t.	n.t.	n.t.	n.t.	n.t.	[73]

^1^ all animals were PERV-A and PERV-B positive, ^2^ the animals were in addition negative for PCV4 and PPV1; ^3^ n.t., not tested; ^4^ the animals were in addition negative for the suid herpesvirus-1 (SuHV-1), 1/10 positive for the porcine reproductive and respiratory syndrome virus (PRRSV), 1/10 positive for the porcine epidemic diarrhea virus (PEDV), negative for the African swine fever virus, and the classical swine fever virus (CSFV); ^5^ PERV proteins were found in different organs by immunohistochemistry; ^6^ strain Troll.

## Data Availability

All data supporting the reported results can be found in this manuscript and in the supplementary materials.

## Supplementary Materials

The following are available online at https://www.mdpi.com/article/10.3390/microorganisms9122617/s1, Figure S1: Workflow of using gBlock gene fragments as the standard for the determination of the copy number, using gBlock I as example, Table S1: gBlock gene fragments used in this study, Table S2: Experimental details concerning quality control criteria when applying a log10 dilutional series of the gBlock in the respective qPCR approaches. Table S3: Virus-specific oligo sequences of the amplicons integrated in gBlocks I, II, III and IV.
