# Supplementary material for: Virological and Parasitological Characterization of Mini-LEWE Minipigs Using Improved Screening Methods and an Overview of Data on Various Minipig Breeds
Source: Microorganisms. 2021 Dec 18;9(12):2617. doi: 10.3390/microorganisms9122617 (PMC8706741; doi:10.3390/microorganisms9122617)
Supplement: Supplementary file 1 [file microorganisms-09-02617-s001.zip › microorganisms-1494674-supplementary.pdf]

Supplementary materials

Table S1 gBlock gene fragments used in this study

gBlock No.	Gene of interest	sequence length	DNA content (in ng/ µl)	copy No. of stock solution
I	PPV-1<br>PCMV<br>PLHV-3<br>HEV<br>PCV3	499 bp	1,69	3,3 x 10^9 copies/ µl
II	PLHV-1<br>PLHV-2<br>PCV4<br>SARS-CoV-2<br>TTSuV-1<br>TTSuV-2	653 bp	2,62	3,9 x 10^9 copies/ µl
III	pGAPDH<br>huGAPDH<br>PERVpol<br>PCV2	690 bp	1,13	1,6 x 10^9 copies/ µl
V	PCV1<br>PCV2<br>PCV3<br>PCV4<br>PLHV-3	623 bp	4,72	7,4 x 10^9 copies/ µl

Table S2: Experimental details concerning quality control criteria when applying a log_{10} dilutional series of the gBlock in the respective qPCR approaches

PCR assay	reference	internal control	PCR efficiency[1]
HEV	[4]	Flu (NA)	0.90
PCMV	[2]	pGAPDH	1.07
PLHV-1	[3]	pGAPDH	1.01
PLHV-2	[3]	pGAPDH	1.01
PLHV-3	[13]	pGAPDH	1.00
PCV1	[12]	pGAPDH	0.90
PCV2	[12]	pGAPDH	1.09
PCV3	[12]	pGAPDH	0.95
PCV4	[12]	pGAPDH	0.85
PPV-1	[1]	pGAPDH	0.94
PERV pol	[11]	pGAPDH	0.95

[1]PCR efficiency curve was calculated with the qPCRsoft software from Analytik Jena (Jena, Germany) and the PCR efficiency was determined as the slope R^2

Table S3 Virus-specific oligosequences integrated in gBlocks I, II, III and IV

GOI*	reference	Oligosequence of the amplicon (5' → 3')
**gBlock I**		
PPV-1	[1]	CAGAATCAGCAACCTCACCACCAACCAAAATATATAATAATGATCTAACT GCAAGCTTAATGGTCGCACTAGACACCAATAACACACTTCCATACACACC AGCAGC
PCMV	[2]	ACTTCGTCGCAGCTCATCTGAGAGAGCTCGACCGCCGCCCTGGCAACCTC GGAATCCCAGAAC
PLHV-3	[3]	AAGGACCCCAAAGAGGAAAATCAATTTTATGGTTCACCTTCTACCTTTCC TTACAGAGTATGCAGTGCCTCAG
HEV	[4]	GGTGGTTTCTGGGGTGACCGGGCTGATTCTCAGCCCTTCGCAATCCCCTA TATTCATCCAACCAACCCCT
PCV3	[5]	AGTGCTCCCCATTGAACGGTGGGGTCATATGTGTTGAGCCATGGGGTGG GTCTGGAGAAAAAGAAGAGGCTTTGTCCTGGGTGAGCGCTGGTAGTTCCC GCCAGAAGTGGTTTGGGGGTGAAGTAACGGCTGTGT
**gBlock II**		
PLHV-1	[3]	CTCACCTCCAAATACAGCGACCTGGTCTACTGAATCGCCGCTAACAGGTC ACTATGGAACACACGATTCAAGC
PLHV-2	[3]	GTCACCTGCAAATACACAGGCCTGGTCTACTGAAGCGCTGCCAATAGGTC AATATGGAACATACGATTCAAGCC
PCV4	[6]	CAGCGACCTTAAAGCGGCTGTGGCCGCCCTGAATGCCGGCAGCTCAATGA GTGAAGTGGCCCGTGAGTTCCCGTCTGTATTTATAAGGTATGGGCGTGGC CTCCGGGACTACGTCATTACTGC
SARS-CoV-2	[7]	ACAGGTACGTTAATAGTTAATAGCGTACTTCTTTTTCTTGCTTTCGTGGT ATTCTTGCTAGTTACACTAGCCATCCTTACTGCGCTTCGATTGTGTGCGT ACTGCTGCAATAT

TTSuV-1	[8]	CGAATGGCTGAGTTTATGCCGCCAGCGGTAGACAGAACTGTCTAGCGAC TGGGCGGGTGCCGGAGGATCCCTGATCCGGAGTCAAGGGGCCTATC
TTSuV-2	[8]	CGAATGGCTGAGTTTATGCCGCTGGTGGTAGACACGAACAGAGCTGAGT GTCTAACCGCCTGGGCGGGTGCCGGAGCTCCTGAGAGCGGAGTCAAGGG GCTTATC
**gBlock III**		
pGAPDH	[9]	ACATGGCCTCCAAGGAGTAAGAGCCCCTGGACCACCAACCCCAGCAAGAG CACGCGAGGAGGAGAGAGGCCCTCAGCTGCTGGGGAGTCACAGCCCCAAC TCGATC
huGAPDH	[10]	GGCGATGCTGGCGCTGAGTACGTCGTGGAGTCCACTGGCGTCTTCACCAC CATGGAGAAGGCTGGGGCTCATTTGCAGGGGGGAGCCAAAAGGGTCATCA TCTCTGCCCCCTCTGCTGATGCCCCCATGTTCGTCATGGGTGTGGACCA
PERV pol	[11]	CGACTGCCCCAAGGGTTCAAGAACTCCCCGACCATCTTTGACGAAGCCCT ACACAGGGACCTGGCCAACTTCAGGATCCAACACCCTCAGGTGACCCTCC TCCAGTACGTGGATGACCTGCTTCTGGCGGGAGCCACCAAACAGGACTGC TTAGAAGGTACGAAGGCACTACTGCTGGAATTGTCTGACCTAGGCTACAG AGCCTCTGCTAAGAAGGCCCAGATTTGCAGGAGAGA
PCV2	[12]	CGGATATTGTATTCCTGGTCGTATATACTGTTTTCGAACGCAGTGCCTAG GCCTACGTGGTCTACATTTCCAGCAGTTTGTAGTCTCAGCCATAGCTGAT TTCTTTTGTTGTTTGGTTGGAAGTAATCAATAGTGGAATCTAGGACAGG

Table S2 (Continuation)

GOI	reference	Oligosequence of the amplicon (5' → 3')
**gBlock IV**		
PCV1	[12]	AACCCCATAAGAGGTGGGTGTTCACCCTTAATAATCCTTCCGAGGAGGAG AAAAACAAAATACGGGAGCTTCCAATCTCCCTTTTTGATTATTTTGTTTG CGGAGAGGAAGGTTTGGAAGAGGGTAGAA
PCV2	[12]	CTGAGTCTTTTTTATCACTTCGTAATGGTTTTTATTATTCACTTAGGGTT AAGTGGGGGGTCTTTAAGATTAAATTCTCTGAATTGTACATACATGGTTA TACGGATATTGTAATCCTGGTCGTATATACTGTTTTCGAACGCAGT
PCV3	[12]	CATAAATGCTCCAAAGCAGTGCTCCCCATTGAACGGTGGGGTCATATGTG TTGAGCCATGGGGTGGGTCTGGAGAAAAAGAAGAGGCTTTGTCCTGGGTGA
PCV4	[12]	ATTATTAAACAGACTTTATTTGTGTCATCACTTCGGATACTACACTTGAT CTTAGCCAAAAGGCTCGTTGATTGAGTGATCACTACGCATTATCCCTGT
PLHV-3	[13]	AACAGCGCCAGAAAAAAAGGACCCCAAAGAGGAAAATCAATTTTATGGTT CACCTTCTACCTTTCC

*GOI = gene of interest

Figure S1 Workflow of using gBlock gene fragments as standard for the determination of the copy number using gBlock I as example

(1) Determination of the genes of interest

(2) Design of a gBlock

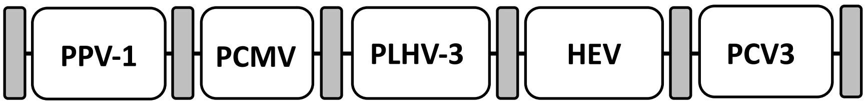

(3) Calculation of the copy number

$$copy\ number\ per\ \mu l\ = c\ x\ M\ x\ 1\ x\ 10^{-15}\ \frac{mol}{fmol}\ x\ \text{Avogadro's number}$$

(4) Preparation of a $\log_{10}$ dilutional series

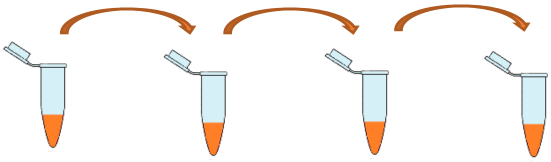

(5) Comparative analysis of the qPCR assays using the $\log_{10}$ dilutional series of the gBlock

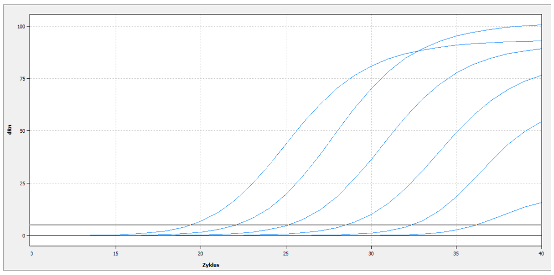

1. Opriessnig, T.; Shen, H.G.; Pal, N.; Ramamoorthy, S.; Huang, Y.W.; Lager, K.M.; Beach, N.M.; Halbur, P.G.; Meng, X.J. A live-attenuated chimeric porcine circovirus type 2 (PCV2) vaccine is transmitted to contact pigs but is not upregulated by concurrent infection with porcine parvovirus (PPV) and porcine reproductive and respiratory syndrome virus (PRRSV) and is efficacious in a PCV2b-PRRSV-PPV challenge model. *Clin Vaccine Immunol* **2011**, *18*, 1261-1268, doi:10.1128/CVI.05057-11.
2. Mueller, N.J.; Barth, R.N.; Yamamoto, S.; Kitamura, H.; Patience, C.; Yamada, K.; Cooper, D.K.; Sachs, D.H.; Kaur, A.; Fishman, J.A. Activation of cytomegalovirus in pig-to-primate organ xenotransplantation. *J Virol* **2002**, *76*, 4734-4740, doi:10.1128/jvi.76.10.4734-4740.2002.
3. Chmielewicz, B.; Goltz, M.; Franz, T.; Bauer, C.; Brema, S.; Ellerbrok, H.; Beckmann, S.; Rziha, H.J.; Lahrmann, K.H.; Romero, C., et al. A novel porcine gammaherpesvirus. *Virology* **2003**, *308*, 317-329, doi:10.1016/s0042-6822(03)00006-0.
4. Jothikumar, N.; Cromeans, T.L.; Robertson, B.H.; Meng, X.J.; Hill, V.R. A broadly reactive one-step real-time RT-PCR assay for rapid and sensitive detection of hepatitis E virus. *J Virol Methods* **2006**, *131*, 65-71, doi:10.1016/j.jviromet.2005.07.004.
5. Palinski, R.; Pineyro, P.; Shang, P.; Yuan, F.; Guo, R.; Fang, Y.; Byers, E.; Hause, B.M. A Novel Porcine Circovirus Distantly Related to Known Circoviruses Is Associated with Porcine Dermatitis and Nephropathy Syndrome and Reproductive Failure. *J Virol* **2017**, *91*, doi:10.1128/JVI.01879-16.
6. Zhang, H.H.; Hu, W.Q.; Li, J.Y.; Liu, T.N.; Zhou, J.Y.; Opriessnig, T.; Xiao, C.T. Novel circovirus species identified in farmed pigs designated as Porcine circovirus 4, Hunan province, China. *Transbound Emerg Dis* **2020**, *67*, 1057-1061, doi:10.1111/tbed.13446.
7. Corman, V.M.; Landt, O.; Kaiser, M.; Molenkamp, R.; Meijer, A.; Chu, D.K.; Bleicker, T.; Brunink, S.; Schneider, J.; Schmidt, M.L., et al. Detection of 2019 novel coronavirus (2019-nCoV) by real-time RT-PCR. *Euro Surveill* **2020**, *25*, doi:10.2807/1560-7917.ES.2020.25.3.2000045.
8. Xiao, C.T.; Gimenez-Lirola, L.; Huang, Y.W.; Meng, X.J.; Halbur, P.G.; Opriessnig, T. The prevalence of Torque teno sus virus (TTSuV) is common and increases with the age of growing pigs in the United States. *J Virol Methods* **2012**, *183*, 40-44, doi:10.1016/j.jviromet.2012.03.026.
9. Duvigneau, J.C.; Hartl, R.T.; Groiss, S.; Gemeiner, M. Quantitative simultaneous multiplex real-time PCR for the detection of porcine cytokines. *J Immunol Methods* **2005**, *306*, 16-27, doi:10.1016/j.jim.2005.06.021.
10. Behrendt, R.; Fiebig, U.; Norley, S.; Gurtler, L.; Kurth, R.; Denner, J. A neutralization assay for HIV-2 based on measurement of provirus integration by duplex real-time PCR. *J Virol Methods* **2009**, *159*, 40-46, doi:10.1016/j.jviromet.2009.02.024.
11. Yang, L.; Guell, M.; Niu, D.; George, H.; Lesha, E.; Grishin, D.; Aach, J.; Shrock, E.; Xu, W.; Poci, J., et al. Genome-wide inactivation of porcine endogenous retroviruses (PERVs). *Science* **2015**, *350*, 1101-1104, doi:10.1126/science.aad1191.
12. Chen, N.; Xiao, Y.; Li, X.; Li, S.; Xie, N.; Yan, X.; Li, X.; Zhu, J. Development and application of a quadruplex real-time PCR assay for differential detection of porcine circoviruses (PCV1 to PCV4) in Jiangsu province of China from 2016 to 2020. *Transbound Emerg Dis* **2021**, *68*, 1615-1624, doi:10.1111/tbed.13833.
13. McMahon, K.J.; Minihan, D.; Campion, E.M.; Loughran, S.T.; Allan, G.; McNeilly, F.; Walls, D. Infection of pigs in Ireland with lymphotropic gamma-herpesviruses and relationship to postweaning multisystemic wasting syndrome. *Vet Microbiol* **2006**, *116*, 60-68, doi:10.1016/j.vetmic.2006.03.022.
